# Local treatment for triple-negative breast cancer patients undergoing chemotherapy: breast-conserving surgery or total mastectomy?

**DOI:** 10.1186/s12885-021-08429-9

**Published:** 2021-06-19

**Authors:** Leqian Guo, Guilan Xie, Ruiqi Wang, Liren Yang, Landi Sun, Mengmeng Xu, Wenfang Yang, Mei Chun Chung

**Affiliations:** 1 Department of Obstetrics and Gynecology, Maternal & Child Health Center, The First Affiliated Hospital of Xi’an Jiaotong University, 277 West Yanta Road, Xi’an, 710061 Shaanxi People’s Republic of China; 2 School of Public Health, Xi’an Jiaotong University Health Science Center, Xi’an, Shaanxi People’s Republic of China; 3 Department of Public Health and Community Medicine, Tufts University School of Medicine, Boston, MA USA

**Keywords:** Triple negative breast cancer, Breast-conserving surgery, Total mastectomy, Radiotherapy, SEER

## Abstract

**Background:**

Because there is no exact therapeutic target, the systemic treatment of triple-negative breast cancer (TNBC) still relies on chemotherapy. In terms of local treatment, based on the highly malignant characteristics of TNBC, it is still uncertain whether patients should be given more aggressive local treatment.

**Methods:**

This study was based on the SEER database. 13,262 TNBC patients undergoing chemotherapy were included. According to local treatment methods, patients were divided into breast-conserving surgery with radiotherapy (BCS + RT), total mastectomy alone and total mastectomy with radiotherapy (Mastectomy+RT). Kaplan-Meier survival analysis drew the survival curves of Overall Survival (OS) and Breast Cancer Specific Survival (BCSS), and Cox proportional risk regression models were used to analyze the impact of different local treatments on OS and BCSS.

**Results:**

After adjusting confounding factors, Mastectomy alone group (*HR* = 1.57; 95%*CI*: 1.40–1.77) and Mastectomy+RT group (*HR* = 1.28; 95%*CI*: 1.12–1.46) were worse in OS than BCS + RT group, and Mastectomy+RT group (*HR* = 0.81; 95%*CI*: 0.73–0.91) was better in OS than Mastectomy alone group. The effect of local treatment for BCSS was similar to that of OS. After stratification according to age, tumor size and lymph node status, when the age was less than 55 years old, at T4, N2 or N3 category, there was no statistical significance between the BCS + RT group and the Mastectomy+RT group in OS or BCSS (all *P* > 0.05). When the age was less than 65 years old, at T1, T2 or N0 category, there was no statistical significance between the Mastectomy alone group and the Mastectomy+RT group in OS or BCSS (all *P* > 0.05). The results of other stratified analyses were basically consistent with the results of total population analysis.

**Conclusion:**

The survival benefit of breast-conserving surgery with radiotherapy was higher than or similar to that of total mastectomy TNBC patients.

## Background

Recently, the International Agency for Research on Cancer (IARC) of the World Health Organization released the latest global cancer data for 2020. The data showes that the incidence of breast cancer in women accounts for 11.7%(2.26 million/1929 million) of new cancer cases in 2020, and has surpassed that of lung cancer, becoming the most common cancer in the world [1]. Triple-negative breast cancer (TNBC) refers to a subtype of breast cancer that is negative for estrogen receptor (ER), progesterone receptor (PR) and HER-2 expression. TNBC accounts for approximately 10–20% of all breast cancer patients [2] and is highly heterogeneous [3]. For example, Lehmann et al. [4] proposes four types based on differences in gene expression profiles of TNBC patients, including basal-like type 1, basal-like type 2, interstitial type and androgen receptor type, and each type showed significant differences in initial symptoms and recurrence patterns. Compared with other breast cancer subtypes, TNBC has unique biological behavior and clinicopathological characteristics. For example, TNBC patients have an earlier onset age, higher aggressiveness, and are more prone to distant metastasis [5–7]. In addition, the median survival time is significantly shorter than other breast cancer subtypes [8]. Due to the lack of expression of ER, PR and HER-2, endocrine therapy and anti-HER-2 targeted therapy for TNBC patients are ineffective, so currently chemotherapy is still the main method of systemic treatment [9]. Although TNBC is sensitive to chemotherapy at the initial stage, the risk of recurrence within 3–5 years after adjuvant treatment is higher than that of other subtypes, so combined local treatment is essential to reduce tumor burden [5].

Surgery is one of the important local treatments for breast cancer patients. Surgical treatment can remove tumor lesions and achieve the goal of radical treatment of breast cancer to a certain extent. According to the recommendations of the 2021 National Comprehensive Cancer Network (NCCN) breast cancer guidelines, in principle, for patients undergoing breast-conserving surgery, regardless of the status of lymph node metastasis, postoperative radiotherapy is recommended; For breast cancer patients undergoing total mastectomy, controversies still exist with respect to the use of radiotherapy [10]. At present, breast-conserving surgery combined with postoperative radiotherapy and total mastectomy (with or without postoperative radiotherapy) have become the most important local treatment for breast cancer patients [11]. Agarwal et al. [12] and Simone et al. [11] conduct some long-term randomized clinical trials over several decades that found no statistically significant difference in long-term survival between breast-conserving combined with postoperative radiotherapy and total mastectomy for early breast cancer patients. Maaren et al. [13] even found a greater survival benefit with breast-conserving combined with postoperative radiotherapy. However, scholars rarely conduct further analysis and discussion on the molecular subtypes of breast cancer in these large-scale clinical trials [11–13]. At present, scholars have found that among T1–2N0 TNBC patients, the breast-conserving surgery combined with postoperative radiotherapy group has a lower 5-year risk of recurrence, but the overall survival rate is similar to that of the total mastectomy patient [14]. Based on this, researchers believe that TNBC should not be a contraindication to breast-conserving therapy. However, the current research results on the local treatment of TNBC are limited, and a unified consensus has not yet been reached [14]. In addition, due to the limitation of sample size and the difference in population race, there is also controversy about whether patients with TNBC need radiotherapy after total mastectomy [15, 16].

Because the breast is the second sex feature of women, breast cancer has its special characteristics compared to other cancers. The appropriate treatment for breast cancer not only causes a great impact on the patient’s body, but also to the patient’s mental health and social interaction [17]. The current treatment of breast cancer is individualized precise treatment based on the molecular subtypes of breast cancer. The present study uses the large number of TNBC cases accumulated in the Surveillance, Epidemiology and End Results (SEER) database to study survival differences between different treatments, which may overcome the shortcomings of previous studies. Furthermore, there are very few relevant studies on further exploring local treatment in the chemotherapy population of TNBC. Therefore, this study retrospectively compared the effects of three local treatments (breast-conserving surgery with postoperative radiotherapy, total mastectomy alone and total mastectomy with postoperative radiotherapy) on the survival and prognosis of patients with TNBC, aiming to provide a certain theoretical basis for the clinical treatment.

## Methods

### Study population

Based on the SEER (Surveillance, Epidemiology and End Results) database, women diagnosed with TNBC from January 2010 to December 2015 were enrolled in this study. Established in 1973 by the National Cancer Institute (NCI), the SEER database is one of the most representative large-scale tumor registry database in North America, covering about 30% of the population of the United States. The database has collected a large number of evidence-based medicine related data, and updated the follow-up data every year, providing systematic evidence support and valuable first-hand information for clinicians’ evidence-based practice and clinical medicine research.

**Fig. 1:**
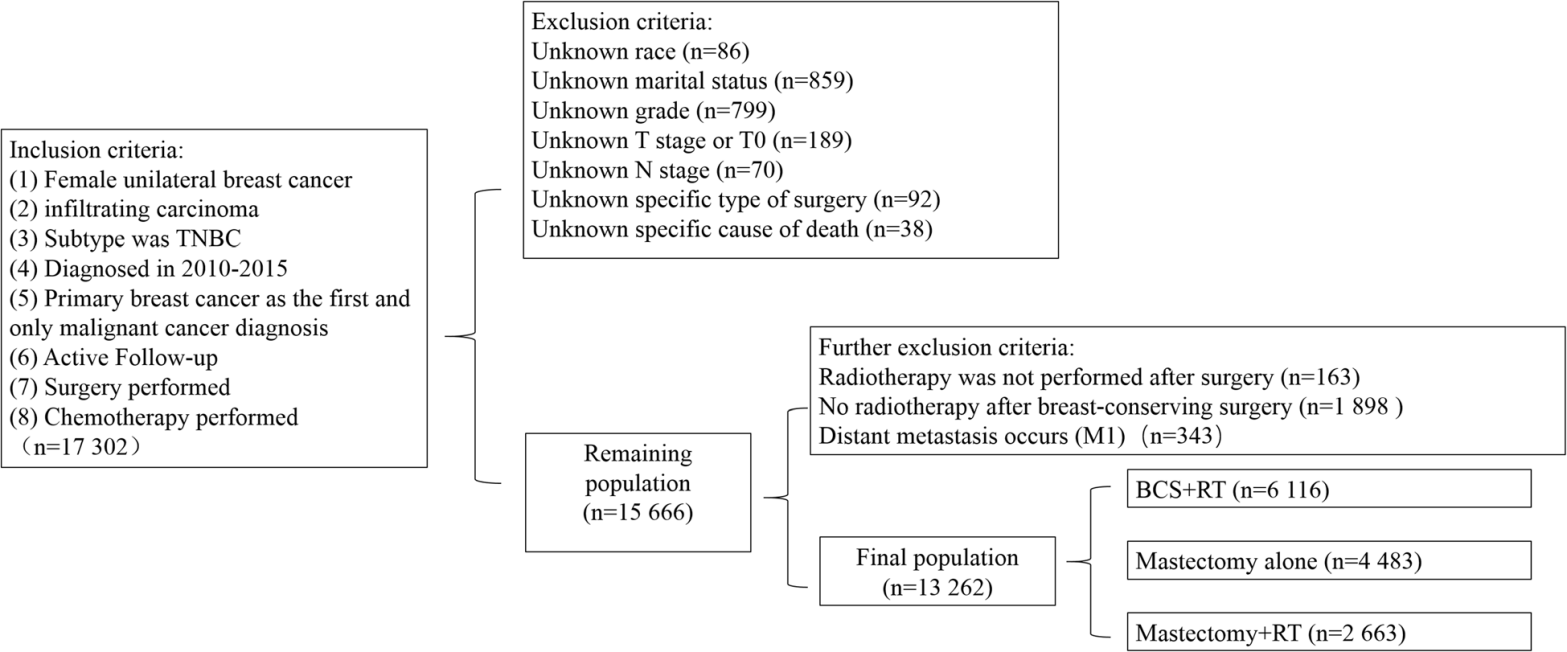
Inclusion and exclusion criteria for this study

Inclusion criteria for this study were: (1) unilateral breast cancer in women; (2) infiltrating carcinoma as the histology type (International Classification of Disease, 3rd edition, ICD-O-3 code was 8500/3) [18]; (3) The molecular subtype of breast cancer was triple negative breast cancer (absence of ER, PR and HER-2); (4) The years of diagnosis were January 2010 to December 2015 (HER-2 receptors had been recorded in the database since 2010); (5) primary breast cancer as the first and only malignant cancer diagnosis; (6) active follow-up; (7) surgery performed; (8) chemotherapy performed. Exclusion criteria were: (1) race, marriage, grade, T category (AJCC 7th) and N category (AJCC 7th) was unknown; (2) There was no surgical information or chemotherapy information and surgery or chemotherapy had not been performed; (3) There was no radiotherapy after breast-conserving surgery; (4) distant metastasis occurs (M1). There were 13,262 patients who met the study criteria (Fig. 1). The data in the SEER database do not require informed patient consent because this database is publicly available and all authors have signed the data-use agreement for the SEER database before accessing the raw data from the SEER database.

### Patients grouping

According to local treatment methods, patients were divided into breast-conserving surgery with postoperative radiotherapy (BCS + RT) group, total mastectomy alone group and total mastectomy with postoperative radiotherapy (Mastectomy+RT) group. The age at diagnosis was divided into < 45, 45 ~ 54, 55 ~ 64 and ≥ 65 years old. Marital status was divided into married and non-married, in which “non-married” included divorce, separation, single, unmarried or cohabiting, widowed [19]; Race was divided into white, black and others, with “others” including American Indians, Alaskan natives, Asians and Pacific Islanders [20, 21]. Laterality was divided into left and right. Primary tumor Site was divided into central portion, upper-inner quadrant, lower-inner quadrant, upper-outer quadrant, lower-outer quadrant, axillary tail, overlapping lesion and others, where, “others” included nipple and unknown location [18, 19]; Tumor histologic grade according to SEER database records was divided into grade I/IIand III/IV; Tumor size (T) and lymph node states (N) were grouped according to AJCC 7th.

### Observation indexes

The main endpoints of this study were Overall Survival (OS) and Breast cancer-specific Survival (BCSS). Among them, OS referred to the survival time of a patient from the time of breast cancer diagnosis to death from any cause (including breast cancer) or to the end of follow-up, and BCSS referred to the survival time of a patient from the time of breast cancer diagnosis to death from breast cancer.

### Statistical methods

R 3.5.1 and R package “survminer”,“survival” (http://www.r-project.org/) were used for statistical analysis in this study. Demographic and clinical pathological characteristics of three groups of people were represented by the number of cases (n) and the percentage (%) and analyzed by chi-square test. Follow-up time was indicated by the median and interquartile interval M(Q1-Q3). Kaplan-Meier (KM) survival analysis was used to draw a survival curve of OS and BCSS for different therapy methods, and Log-rank test was used to calculate the significance of each group. Univariate and multivariate prognostic analyses were performed using Cox proportional risk regression model. Hazard Ratio (*HR*) and 95% Confidence Interval (*CI*) were calculated. In addition, since age at diagnosis, tumor size and lymph node metastasis of patients had an impact on the choice of treatment modality and survival prognosis of patients, we performed stratified analyses based on age at diagnosis, tumor size and lymph node metastasis to verify the stability of the model. We considered that the difference was statistically significant when *P* < 0.05.

## Results

### Patient demographic and clinical characteristics

A total of 13,262 patients were included in this study. They were divided into three groups according to local treatment methods: 6116(46.12%) patients in the breast-conserving surgery with postoperative radiotherapy (BCS + RT) group, 4483(33.80%) patients in the total mastectomy alone group, and 2663(20.08%) patients in the total mastectomy with postoperative radiotherapy (Mastectomy+RT) group. There were statistically significant differences in age at diagnosis, ethnicity, primary tumor site, grade, tumor size and lymph node status among the three groups (*χ*^2^ values were 507.07, 59.70, 375.59, 11.78, 2086.50 and 2575.07, respectively, all *P* < 0.05). Patients with older, black, located in the axillary tail, lower tumor grade, smaller tumor and less lymphatic metastasis were more likely to be treated with radiotherapy after breast-conserving surgery (Table 1).

**Table 1:** Basic characteristics of all TNBC patients [n(%)]

Variable	Group	All patients (*n* = 13,262)	BCS + RT (*n* = 6116)	Mastectomy alone (*n* = 4483)	Mastectomy+RT (*n* = 2663)	*χ*^2^	*P*
Age	< 45	3067(23.13)	918(15.01)	1312(29.27)	837(31.43)	507.07	0.000
	45 ~ 54	3854(29.06)	1748(28.58)	1326(29.58)	780(29.29)		
	55 ~ 64	3637(27.42)	1973(32.26)	1039(23.18)	625(23.47)		
	≥65	2704(20.39)	1477(24.15)	806(17.98)	421(15.81)		
Ethnicity	White	9392(70.82)	4279(69.96)	3295(73.50)	1818(68.27)	59.70	0.000
	Black	2855(21.53)	1426(23.32)	807(18.00)	622(23.36)		
	Others ^a^	1015(7.65)	411(6.72)	381(8.50)	223(8.37)		
Marital status	non-Married ^b^	5169(38.98)	2332(38.13)	1763(39.33)	1074(40.33)	4.13	0.127
	Married	8093(61.02)	3784(61.87)	2720(60.67)	1589(59.67)		
Laterality	Left	6767(51.03)	3188(52.13)	2243(50.03)	1336(50.17)	5.51	0.064
	Right	6495(48.97)	2928(47.87)	2240(49.97)	1327(49.83)		
Primary tumor Site	Central portion	407(3.07)	120(1.96)	156(3.48)	131(4.92)	375.59	0.000
	Upper-inner quadrant	1864(14.06)	950(15.53)	656(14.63)	258(9.69)		
	Lower-inner quadrant	808(6.09)	405(6.62)	278(6.20)	125(4.69)		
	Upper-outer quadrant	5222(39.38)	2555(41.78)	1634(36.45)	1033(38.79)		
	Lower-outer quadrant	936(7.06)	426(6.97)	325(7.25)	185(6.95)		
	Axillary tail	96(0.72)	54(0.88)	26(0.58)	16(0.60)		
	Overlapping lesion	2919(22.01)	1366(22.33)	990(22.08)	563(21.14)		
	Others ^c^	1010(7.62)	240(3.92)	418(9.32)	352(13.22)		
Grade	I/II	1813(13.67)	874(14.29)	629(14.03)	310(11.64)	11.78	0.003
	III/IV	11,449(86.33)	5242(85.71)	3854(85.97)	2353(88.36)		
Tumor size	T1	5328(40.17)	3222(52.68)	1685(37.59)	421(15.81)	2086.50	0.000
	T2	6138(46.28)	2629(42.99)	2231(49.77)	1278(47.99)		
	T3	1216(9.17)	218(3.56)	381(8.50)	617(23.17)		
	T4	580(4.37)	47(0.77)	186(4.15)	347(13.03)		
Lymph node status	N0	8095(61.04)	4500(73.58)	3041(67.83)	554(20.80)	2575.07	0.000
	N1	3619(27.29)	1284(20.99)	1070(23.87)	1265(47.50)		
	N2	937(7.07)	226(3.70)	217(4.84)	494(18.55)		
	N3	611(4.61)	106(1.73)	155(3.46)	350(13.14)		

*Abbreviations*: *BCS* breast-conserving surgery, *RT* radiotherapy

^a^Including American Indians, Alaskan natives, Asians and Pacific Islanders

^b^Including divorce, separation, single, unmarried or cohabiting, widowed

^c^Including nipple and unknown location

### Survival analysis

**Fig. 2:**
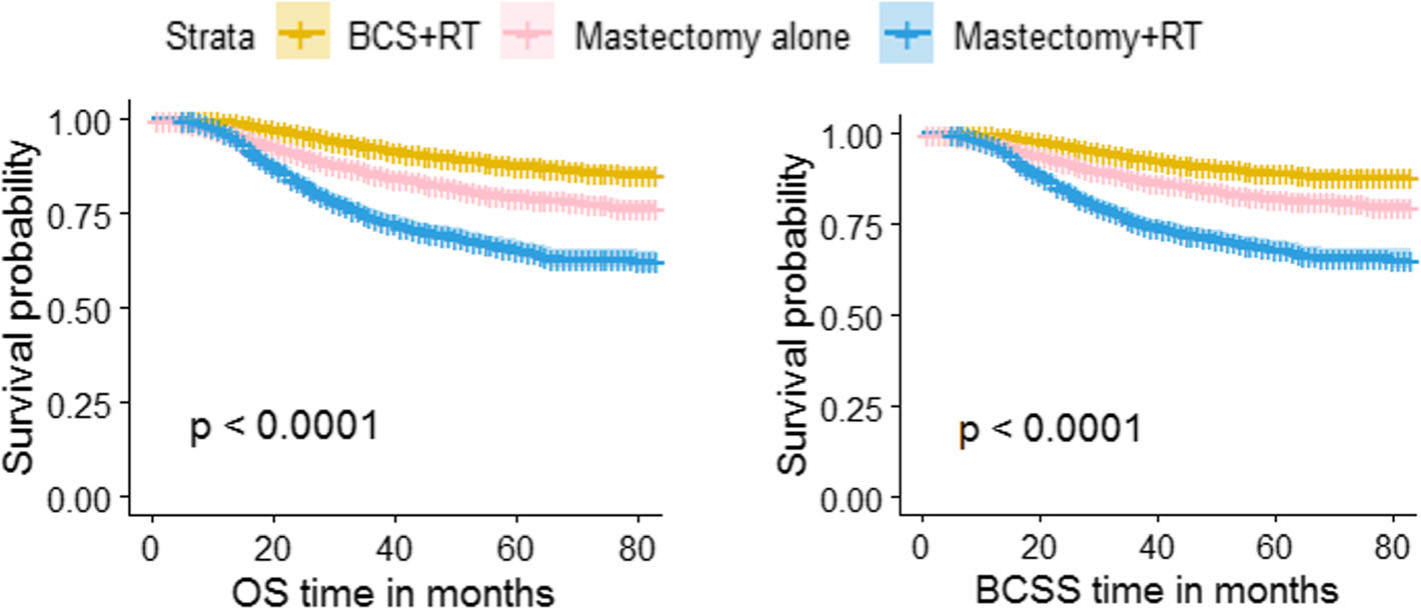
Kaplan-Meier curves for overall survival and breast cancer specific survival by treatment type for all patients

The 3-year OS and 3-year BCSS of all patients in this study were 86.8 and 88.2%, respectively. The 3-year OS of patients who received BCS + RT, Mastectomy alone and Mastectomy+RT were 92.9, 86.0 and 74.3%, respectively, and the 3-year BCSS of patients in the three groups were 93.6, 87.9 and 76.2%, respectively. The 5-year OS and 5-year BCSS of all patients in this study were 80.5 and 82.6%, respectively. The 5-year OS of patients who received BCS + RT, Mastectomy alone and Mastectomy+RT were 87.9, 79.6, and 65.5%, respectively, and the 5-year BCSS of patients in the three groups were 89.2, 82.3, and 68.3%, respectively. Survival analyses showed that patients with TNBC who received BCS + RT had better OS and BCSS (all log-rank *P* < 0.05). From the KM survival curve, we could also observe that the OS curve and BCSS curve of patients in the three groups showed a separation trend (Fig. 2).

**Fig. 3:**
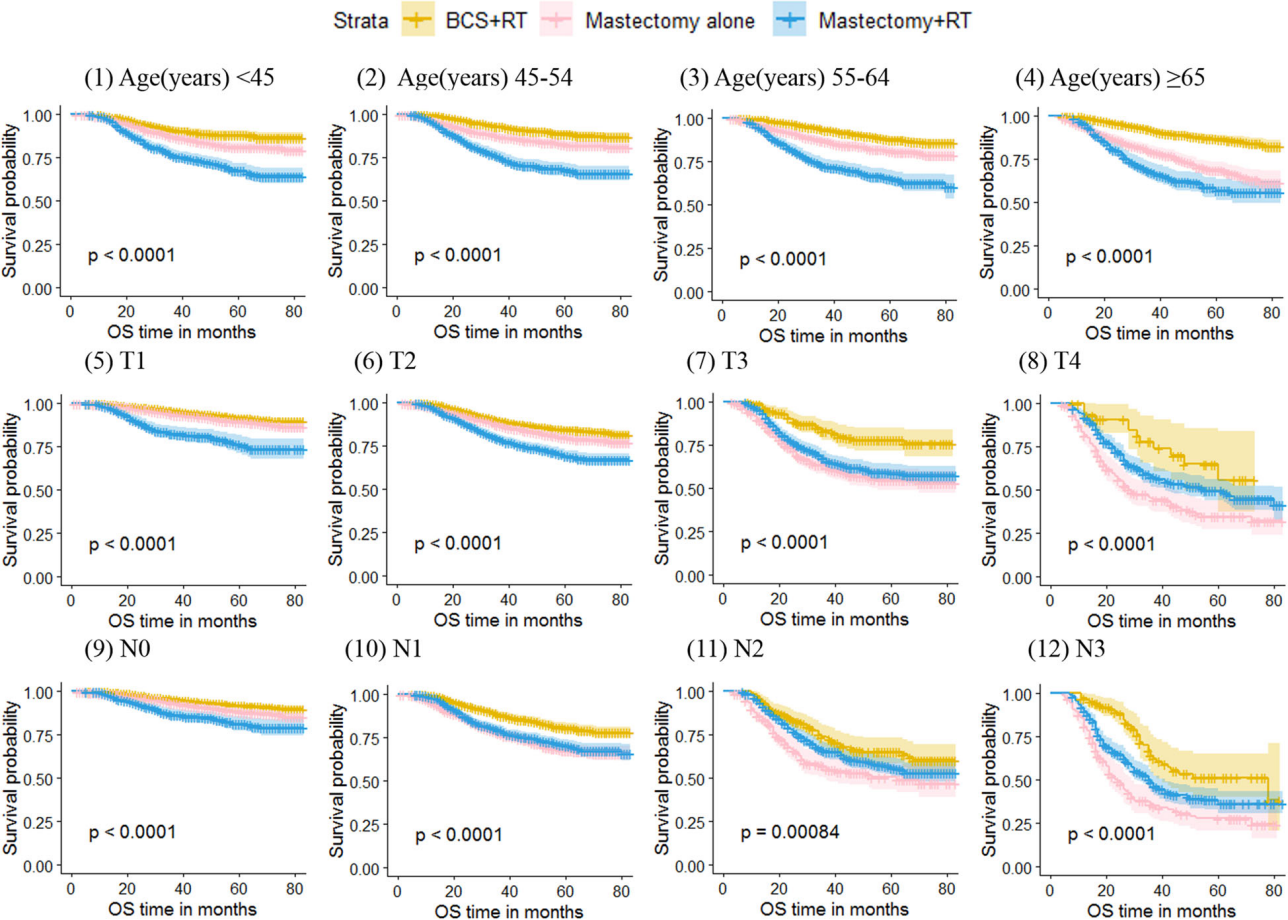
Kaplan-Meier curves of overall survival by treatment type for all patients, stratified by age at diagnosis, tumor size and lymph node status. Patients were aged < 45 years(1),45 to 54 years(2),55 to 64 years(3), and ≥ 65 years(4). Patients were classified as T1(5), T2(6), T3(7) and T4(8) according to tumor size and were classified as N0(9), N1(10), N2(11) and N3(12) according to lymph node status

**Fig. 4:**
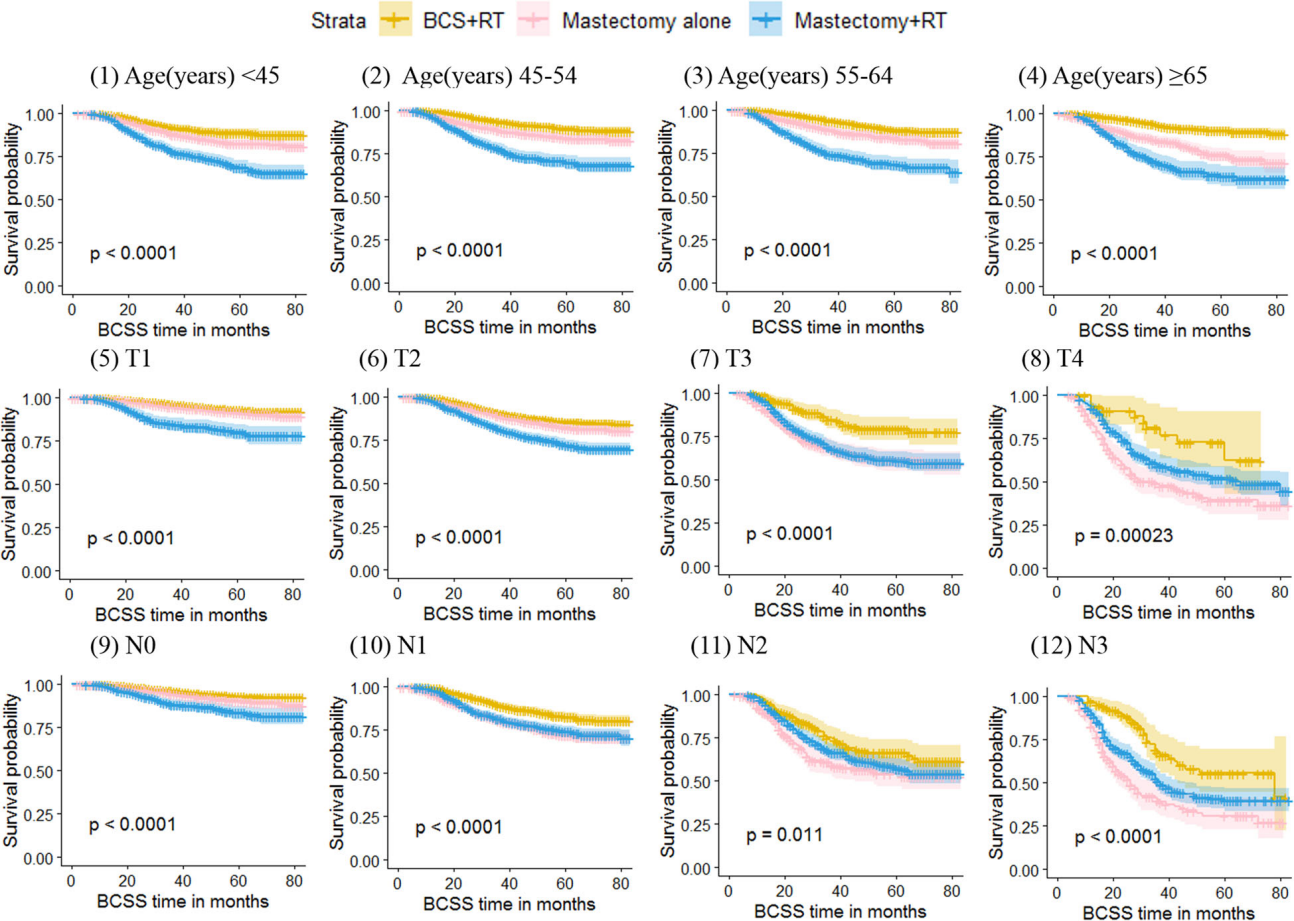
Kaplan-Meier curves of breast cancer specific survival by treatment type for all patients, stratified by age at diagnosis, tumor size and lymph node status. Patients were aged < 45 years(1),45 to 54 years(2),55 to 64 years(3), and ≥ 65 years(4). Patients were classified as T1(5), T2(6), T3(7) and T4(8) according to tumor size and were classified as N0(9), N1(10), N2(11) and N3(12) according to lymph node status

When survival analyses were conducted based on age, the results showed that the OS of patients in the BCS + RT group was the best, and the OS in the Mastectomy+RT group was the worst when the age was less than 65 years old [Fig. 3(1 ~ 4)]. When stratified according to tumor size, the OS in the BCS + RT group was the best at T1, T2 and T3 categories, and the OS in the Mastectomy alone group was the worst at T3 and T4 categories [Fig. 3(5 ~ 8)]. When stratified according to lymph node status, the OS in the BCS + RT group at N0 and N1 categories was the best, and the OS in the Mastectomy alone group was the worst at N1, N2 and N3 categories [Fig. 3(9 ~ 12)]. The BCSS survival curves of the three groups at different ages, tumor sizes and lymph node states were basically consistent with the OS trends (Fig. 4).

### Cox analysis of OS and BCSS among TNBC patients

After adjusting age at diagnosis, ethnicity, marital status, laterality, primary tumor site, grade, tumor size and lymph node status, we constructed a multivariate Cox proportional risk regression model to explore the effect of local treatment methods on overall survival. The C-index and 95% confidence interval of the model was 0.773(0.763–0.783). The results of multivariate Cox proportional risk regression model showed that Mastectomy alone group (*HR* = 1.57; 95%*CI*: 1.40–1.77) and Mastectomy+RT group (*HR* = 1.28; 95%*CI*: 1.12–1.46) were worse in OS than BCS + RT group, and Mastectomy+RT group (*HR* = 0.81; 95%*CI*: 0.73–0.91) was better in OS than Mastectomy alone group (Table 2). In addition, we constructed a multivariate Cox proportional risk regression model to explore the effect of local treatment methods on breast cancer specific survival. The C-index and 95% confidence interval of the model was 0.779(0.769–0.789). The results of multivariate Cox proportional risk regression model showed that Mastectomy alone group (*HR* = 1.49; 95%*CI*: 1.31–1.70) and Mastectomy+RT group (*HR* = 1.24; 95%*CI*: 1.08–1.43) were worse in BCSS than in the BCSS+RT group, and Mastectomy+RT group (*HR* = 0.83; 95%*CI*: 0.74–0.94) was better in OS than Mastectomy alone group (Table 3). After stratification according to age, tumor size and lymph node status, when the age was less than 55 years old, at T4, N2 or N3 category, there was no statistical significance between the BCS + RT group and the Mastectomy+RT group in OS or BCSS (all *P* > 0.05). When the age was less than 65 years old, at T1, T2 or N0 category, there was no statistical significance between the Mastectomy alone group and the Mastectomy+RT group in OS or BCSS (all *P* > 0.05). The results of other stratified analyses were basically consistent with the results of total population analysis (Table 2 and Table 3).

**Table 2:** Cox proportional risk regression analysis of Overall Survival among TNBC patients who underwent BCS or mastectomy

Variable	Group	Reference	Local treatment	Univariable analysis		Multivariable analysis	
				*HR*(95%*CI*)	*P*	*HR*(95%*CI*)	*P*
Total ^a^		BCS + RT	Mastectomy alone	1.94(1.73–2.18)	0.000	1.57(1.40–1.77)	0.000
		BCS + RT	Mastectomy+RT	3.59(3.20–4.02)	0.000	1.28(1.12–1.46)	0.000
		Mastectomy alone	Mastectomy+RT	1.85(1.66–2.05)	0.000	0.81(0.73–0.91)	0.000
Age ^b^	< 45	BCS + RT	Mastectomy alone	1.57(1.21–2.05)	0.001	1.47(1.12–1.92)	0.005
		BCS + RT	Mastectomy+RT	3.00(2.32–3.88)	0.000	1.23(0.94–1.62)	0.139
		Mastectomy alone	Mastectomy+RT	1.91(1.56–2.33)	0.000	0.84(0.68–1.04)	0.109
	45 ~ 54	BCS + RT	Mastectomy alone	1.78(1.42–2.22)	0.000	1.51(1.20–1.91)	0.000
		BCS + RT	Mastectomy+RT	3.63(2.92–4.52)	0.000	1.25(0.98–1.60)	0.072
		Mastectomy alone	Mastectomy+RT	2.05(1.67–2.51)	0.000	0.83(0.66–1.03)	0.095
	55 ~ 64	BCS + RT	Mastectomy alone	1.87(1.50–2.34)	0.000	1.44(1.15–1.82)	0.002
		BCS + RT	Mastectomy+RT	3.93(3.17–4.86)	0.000	1.30(1.01–1.68)	0.040
		Mastectomy alone	Mastectomy+RT	2.10(1.69–2.61)	0.000	0.90(0.71–1.14)	0.396
	≥65	BCS + RT	Mastectomy alone	2.82(2.25–3.52)	0.000	1.99(1.57–2.52)	0.000
		BCS + RT	Mastectomy+RT	4.13(3.25–5.25)	0.000	1.38(1.04–1.82)	0.027
		Mastectomy alone	Mastectomy+RT	1.47(1.17–1.83)	0.001	0.69(0.54–0.88)	0.003
Tumor size ^c^	T1	BCS + RT	Mastectomy alone	1.36(1.08–1.71)	0.009	1.37(1.09–1.74)	0.008
		BCS + RT	Mastectomy+RT	3.54(2.71–4.62)	0.000	1.49(1.10–2.02)	0.011
		Mastectomy alone	Mastectomy+RT	2.60(1.96–3.46)	0.000	1.08(0.79–1.49)	0.615
	T2	BCS + RT	Mastectomy alone	1.36(1.16–1.60)	0.000	1.44(1.22–1.69)	0.000
		BCS + RT	Mastectomy+RT	2.20(1.87–2.59)	0.000	1.27(1.06–1.53)	0.008
		Mastectomy alone	Mastectomy+RT	1.62(1.38–1.90)	0.000	0.88(0.74–1.05)	0.170
	T3	BCS + RT	Mastectomy alone	2.61(1.81–3.77)	0.000	2.21(1.52–3.22)	0.000
		BCS + RT	Mastectomy+RT	2.15(1.51–3.07)	0.000	1.61(1.12–2.31)	0.011
		Mastectomy alone	Mastectomy+RT	0.82(0.66–1.02)	0.082	0.73(0.58–0.91)	0.005
	T4	BCS + RT	Mastectomy alone	2.69(1.48–4.89)	0.001	2.34(1.24–4.41)	0.009
		BCS + RT	Mastectomy+RT	1.73(0.96–3.12)	0.068	1.36(0.73–2.54)	0.334
		Mastectomy alone	Mastectomy+RT	0.64(0.50–0.83)	0.001	0.58(0.45–0.75)	0.000
Lymph node status ^d^	N0	BCS + RT	Mastectomy alone	1.53(1.28–1.83)	0.000	1.44(1.20–1.73)	0.000
		BCS + RT	Mastectomy+RT	2.66(2.06–3.44)	0.000	1.57(1.17–2.11)	0.003
		Mastectomy alone	Mastectomy+RT	1.74(1.35–2.25)	0.000	1.09(0.82–1.45)	0.543
	N1	BCS + RT	Mastectomy alone	1.97(1.63–2.39)	0.000	1.74(1.43–2.12)	0.000
		BCS + RT	Mastectomy+RT	1.76(1.46–2.13)	0.000	1.38(1.13–1.69)	0.002
		Mastectomy alone	Mastectomy+RT	0.89(0.75–1.06)	0.196	0.79(0.67–0.95)	0.009
	N2	BCS + RT	Mastectomy alone	1.83(1.32–2.53)	0.000	1.45(1.03–2.04)	0.034
		BCS + RT	Mastectomy+RT	1.32(0.99–1.76)	0.060	1.06(0.78–1.44)	0.693
		Mastectomy alone	Mastectomy+RT	0.72(0.56–0.93)	0.012	0.73(0.57–0.95)	0.019
	N3	BCS + RT	Mastectomy alone	2.64(1.80–3.88)	0.000	2.10(1.40–3.17)	0.000
		BCS + RT	Mastectomy+RT	1.78(1.24–2.54)	0.002	1.44(0.98–2.10)	0.063
		Mastectomy alone	Mastectomy+RT	0.67(0.52–0.86)	0.002	0.68(0.53–0.88)	0.004

Abbreviations: *BCS* breast-conserving surgery, *RT* radiotherapy, *CI* confidence interval, *HR* hazard ratio

^a^Multivariable analysis adjusted for age at diagnosis, ethnicity, marital status, laterality, primary tumor site, grade, tumor size and lymph node status

^b^Multivariable analysis adjusted for ethnicity, marital status, laterality, primary tumor site, grade, tumor size and lymph node status

^c^Multivariable analysis adjusted for age at diagnosis, ethnicity, marital status, laterality, primary tumor site, grade and lymph node status

^d^Multivariable analysis adjusted for age at diagnosis, ethnicity, marital status, laterality, primary tumor site, grade and tumor size

**Table 3:** Cox proportional risk regression of BCSS among TNBC patients who underwent BCS or mastectomy

Variable	Group	Reference	Local treatment	Univariable analysis		Multivariable analysis	
				*HR*(95%*CI*)	*P*	*HR*(95%*CI*)	*P*
Total^a^		BCS + RT	Mastectomy alone	1.90(1.67–2.15)	0.000	1.49(1.31–1.70)	0.000
		BCS + RT	Mastectomy+RT	3.75(3.33–4.24)	0.000	1.24(1.08–1.43)	0.002
		Mastectomy alone	Mastectomy+RT	1.98(1.77–2.22)	0.000	0.83(0.74–0.94)	0.003
Age^b^	< 45	BCS + RT	Mastectomy alone	1.56(1.19–2.06)	0.001	1.44(1.09–1.91)	0.010
		BCS + RT	Mastectomy+RT	3.10(2.38–4.05)	0.000	1.24(0.93–1.65)	0.135
		Mastectomy alone	Mastectomy+RT	1.99(1.61–2.44)	0.000	0.86(0.69–1.07)	0.186
	45 ~ 54	BCS + RT	Mastectomy alone	1.75(1.38–2.21)	0.000	1.50(1.18–1.90)	0.001
		BCS + RT	Mastectomy+RT	3.61(2.87–4.53)	0.000	1.21(0.94–1.56)	0.145
		Mastectomy alone	Mastectomy+RT	2.06(1.66–2.56)	0.000	0.81(0.64–1.02)	0.073
	55 ~ 64	BCS + RT	Mastectomy alone	1.74(1.37–2.21)	0.000	1.35(1.05–1.73)	0.018
		BCS + RT	Mastectomy+RT	3.87(3.09–4.85)	0.000	1.24(0.95–1.62)	0.112
		Mastectomy alone	Mastectomy+RT	2.22(1.75–2.81)	0.000	0.92(0.71–1.19)	0.522
	≥65	BCS + RT	Mastectomy alone	2.79(2.15–3.62)	0.000	1.87(1.42–2.47)	0.000
		BCS + RT	Mastectomy+RT	4.73(3.60–6.21)	0.000	1.35(0.98–1.86)	0.066
		Mastectomy alone	Mastectomy+RT	1.70(1.32–2.18)	0.000	0.72(0.55–0.95)	0.019
Tumor size^c^	T1	BCS + RT	Mastectomy alone	1.33(1.03–1.72)	0.028	1.31(1.01–1.70)	0.045
		BCS + RT	Mastectomy+RT	3.67(2.74–4.91)	0.000	1.40(1.00–1.96)	0.048
		Mastectomy alone	Mastectomy+RT	2.75(2.01–3.77)	0.000	1.07(0.76–1.52)	0.699
	T2	BCS + RT	Mastectomy alone	1.29(1.08–1.53)	0.004	1.35(1.13–1.60)	0.001
		BCS + RT	Mastectomy+RT	2.19(1.84–2.61)	0.000	1.24(1.02–1.50)	0.029
		Mastectomy alone	Mastectomy+RT	1.71(1.44–2.03)	0.000	0.92(0.76–1.11)	0.374
	T3	BCS + RT	Mastectomy alone	2.41(1.63–3.54)	0.000	2.00(1.35–2.97)	0.001
		BCS + RT	Mastectomy+RT	2.19(1.51–3.17)	0.000	1.58(1.08–2.31)	0.018
		Mastectomy alone	Mastectomy+RT	0.91(0.72–1.15)	0.431	0.79(0.62–1.00)	0.054
	T4	BCS + RT	Mastectomy alone	2.91(1.52–5.59)	0.001	2.62(1.31–5.26)	0.007
		BCS + RT	Mastectomy+RT	1.92(1.01–3.65)	0.046	1.52(0.77–3.02)	0.231
		Mastectomy alone	Mastectomy+RT	0.66(0.51–0.86)	0.002	0.58(0.44–0.76)	0.000
Lymph node status^d^	N0	BCS + RT	Mastectomy alone	1.51(1.24–1.84)	0.000	1.38(1.12–1.69)	0.002
		BCS + RT	Mastectomy+RT	2.81(2.13–3.69)	0.000	1.63(1.19–2.24)	0.002
		Mastectomy alone	Mastectomy+RT	1.86(1.41–2.45)	0.000	1.19(0.88–1.61)	0.268
	N1	BCS + RT	Mastectomy alone	1.87(1.52–2.30)	0.000	1.64(1.32–2.03)	0.000
		BCS + RT	Mastectomy+RT	1.71(1.40–2.10)	0.000	1.31(1.05–1.62)	0.017
		Mastectomy alone	Mastectomy+RT	0.92(0.76–1.10)	0.359	0.80(0.66–0.96)	0.018
	N2	BCS + RT	Mastectomy alone	1.67(1.19–2.35)	0.003	1.30(0.91–1.85)	0.156
		BCS + RT	Mastectomy+RT	1.33(0.99–1.79)	0.058	1.05(0.77–1.44)	0.753
		Mastectomy alone	Mastectomy+RT	0.80(0.61–1.04)	0.096	0.81(0.62–1.06)	0.132
	N3	BCS + RT	Mastectomy alone	2.71(1.81–4.07)	0.000	2.07(1.34–3.19)	0.001
		BCS + RT	Mastectomy+RT	1.86(1.28–2.72)	0.001	1.44(0.96–2.16)	0.077
		Mastectomy alone	Mastectomy+RT	0.69(0.53–0.89)	0.005	0.70(0.53–0.91)	0.008

*Abbreviations*: *BCS* breast-conserving surgery, *RT* radiotherapy, *CI* confidence interval, *HR* hazard ratio

^a^Multivariable analysis adjusted for age at diagnosis, ethnicity, marital status, laterality, primary tumor site, grade, tumor size and lymph node status

^b^Multivariable analysis adjusted for ethnicity, marital status, laterality, primary tumor site, grade, tumor size and lymph node status

^c^Multivariable analysis adjusted for age at diagnosis, ethnicity, marital status, laterality, primary tumor site, grade and lymph node status

^d^Multivariable analysis adjusted for age at diagnosis, ethnicity, marital status, laterality, primary tumor site, grade and tumor size

## Discussion

This study used the SEER database and based on a large sample of clinical data to retrospectively analyze and compare the survival differences of TNBC patients undergoing chemotherapy with three different local treatments. The results found that among 13,262 TNBC patients undergoing chemotherapy, after adjusting for age, race, marital status, laterality, primary tumor site and lymph node status, the OS benefit and BCSS benefit in breast-conserving surgery with radiotherapy group were higher than that of total mastectomy with radiotherapy group and mastectomy alone group. After stratified analysis according to diagnosis age, tumor size and lymph node status, the survival benefit of breast-conserving surgery with radiotherapy group was still higher than or similar to that of total mastectomy patients. It suggested that the model was stable and provided a certain reference basis for clinically formulating the best treatment plan for TNBC.

After stratification by age, the results showed that when the age was less than 55 years, patients with breast-conserving postoperative radiotherapy and total mastectomy with radiotherapy had similar OS and BCSS, but the survival benefit of breast-conserving postoperative radiotherapy was still higher than that of simple total mastectomy. Young breast cancer patients were a unique group, usually with more aggressive tumors and a worse prognosis [22–24]. This study suggested that breast-conserving surgery with radiotherapy was still feasible for young TNBC patients, which was basically consistent with the conclusions of Vila et al. [25]. When the age was older than 55 years, the OS of breast-conserving surgery with radiotherapy group were higher than those of the two total mastectomy groups, suggesting that breast-conserving surgery with postoperative radiotherapy was also safe in the elderly and especially could be considered in the case of elderly patients whose physical condition cannot well withstand total mastectomy. After stratified analysis according to tumor size and lymph node metastasis, the results showed that when the patients were T4, N2 or N3 category, there were similar OS and BCSS between breast-conserving surgery with radiotherapy group and total mastectomy with radiotherapy, but their survival benefits were still higher than that of mastectomy alone. In addition, when the patients were T1, T2 or N0 category, radiotherapy after total mastectomy did not bring higher survival benefits to patients than mastectomy alone, but the survival benefit of radiotherapy after breast-conserving surgery was still the highest. For patients with small lesions and negative lymph nodes, NCCN guidelines did not recommend postoperative radiotherapy for patients undergoing total mastectomy, which was consistent with the results of this study. However, Bhoo-Pathy et al. [26] carried out a large multi-center retrospective study of 775 Asian patients with T1–2, N0–1, M0 TNBC, and found there was no significant difference in survival rates between patients who received only total mastectomy and those who received breast-conserving postoperative radiotherapy, which was slightly different from the results of our study. There may be two reasons to explain the difference. On the one hand, there was a large difference in the sample size between the studies, and the above-mentioned study focused on Asian populations. Racial disparities in TNBC incidence have been well-documented [20, 21, 27]. On the other hand, Bhoo-Pathy et al. [26] conducted the study between 2006 and 2011. With the progress of precision surgery technology, the surgical treatment of breast cancer strives to reduce the operation-related injuries and improve the postoperative function while ensuring the therapeutic effect. Breast-conserving surgery replaces partial total mastectomy in accordance with the principle of least effective treatment. In addition, with the extensive development of breast cancer screening and the continuous improvement of the pathological complete response (pCR) rate of neoadjuvant treatment, the rates of small tumors and untouchable breast cancer are on the rise. In 2014, the American Society of Oncology Surgeons proposed the standard of safe margin for “no ink on tumor” in breast-conserving surgery [28], and this principle continues to be used in the latest NCCN guidelines [10]. Of course, whether patients receiving neoadjuvant chemotherapy can accurately evaluate the pCR rate through minimally invasive biopsy technology is still a research hotspot. In a word, the early diagnosis of tumors, surgical and radiotherapy techniques had been greatly improved in recent years, which may also explained why our study could observe that patients with TNBC who had breast-conserving postoperative radiotherapy had better OS and BCSS.

In addition, we have some suggestions for the future research direction of TNBC. First of all, through genomic and transcriptome analysis of large samples of TNBC patients, Lehmann et al. [4] and Jiang et al. [29] have proposed a “quadrate” of TNBC, which provides a basis for precision treatment of TNBC. However, at present, there is no unified conclusion on TNBC molecular typing. Considering the high cost of gene testing, it is still a key research direction in the future to explore the typing method that consistent with the gene expression profile and based on immunohistochemical indicators. Secondly, patients with TNBC who receive neoadjuvant therapy and achieve pathological complete response have a significantly improved prognosis [30]. Currently, anthracyclines and taxanes are the main neoadjuvant therapy regimens for TNBC [30]. However, with the in-depth understanding of the molecular nature of TNBC, neoadjuvant therapy has been continuously explored and optimized, including the optimization of platinum drugs and the use of immune checkpoint inhibitors, which have achieved good results [31]. Thirdly, with the continuous advancement of breast cancer diagnosis and treatment technology, the survival period of breast cancer patients had been significantly extended. Therefore, it is particularly important to pay attention to the quality of life of patients when the surgical method is judged [32].

This study used the SEER database with a large sample size and strong objectivity of the results. However, the existing problems could not be ignored. It was a retrospective analysis with selection bias. We established Cox multivariate regression models and conducted multiple sensitivity analyses to reduce the confounding bias and make the research results more credible and referential. The SEER database had been recording the expression of HER-2 in breast cancer patients since 2010, and the follow-up time was relatively short. Inadequate follow-up duration may lead to skewed results. Therefore, a longer follow-up was needed to further verify the research conclusions. TNBC was a heterogeneous tumor that contained many subtypes [33, 34]. Whether different subtypes had different clinicopathological characteristics and survival outcomes still needed to be further explored. In addition, the SEER database cannot directly obtain prior surgery and specific chemotherapy information, which cause the bias in the results [30]. In the future, prospective randomized controlled clinical trials should be conducted on the gene level to investigate the therapeutic effects of neoadjuvant chemotherapy on patients.

## Conclusions

Triple-negative breast cancer was a subtype of breast cancer with higher malignancy and poor prognosis. Based on a large sample of clinical data, this study analyzed the survival differences between local treatments in TNBC patients who had undergone chemotherapy. It found that breast-conserving surgery combined with postoperative radiotherapy improved the OS and BCSS of TNBC patients. However, the conclusions of this study have certain limitations, and the results need to be confirmed by more follow-up and more prospective studies.

## Abbreviations

OS: Overall Survival
BCSS: Breast Cancer Specific Survival
SEER: Surveillance, Epidemiology, and End Results
BCS: Breast-conserving Surgery
RT: Radiotherapy
CI: Confidence Interval; *HR*: Hazard Ratio
